# Experiences With VA-Purchased Community Care for US Veterans With Mental Health Conditions

**DOI:** 10.1001/jamanetworkopen.2025.11548

**Published:** 2025-05-21

**Authors:** Megan E. Vanneman, Eric T. Roberts, Yaming Li, Florentina E. Sileanu, Utibe R. Essien, Maria K. Mor, Michael J. Fine, Carolyn T. Thorpe, Thomas R. Radomski, Katie J. Suda, Walid F. Gellad

**Affiliations:** 1 Veterans Affairs (VA) Informatics, Decision Enhancement and Analytic Sciences Center, VA Salt Lake City Health Care System, Salt Lake City, Utah; 2 Department of Internal Medicine, University of Utah School of Medicine, Salt Lake City; 3 Department of Population Health Sciences, University of Utah School of Medicine, Salt Lake City; 4 Division of General Internal Medicine, Perelman School of Medicine, University of Pennsylvania, Philadelphia; 5 VA Center for Health Equity Research and Promotion, Corporal Michael J. Crescenz Department of Veterans Affairs Medical Center, Philadelphia, Pennsylvania; 6 VA Center for Health Equity Research and Promotion, VA Pittsburgh Healthcare System, Pittsburgh, Pennsylvania; 7 David Geffen School of Medicine, University of California, Los Angeles; 8 VA Center for the Study of Healthcare Innovation, Implementation, and Policy, VA Greater Los Angeles Healthcare System, West Los Angeles, California; 9 Department of Biostatistics and Data Science, School of Public Health, University of Pittsburgh, Pittsburgh, Pennsylvania; 10 School of Medicine, University of Pittsburgh, Pittsburgh, Pennsylvania; 11 Eshelman School of Pharmacy, University of North Carolina at Chapel Hill

## Abstract

**Importance:**

Veterans with mental health conditions (MHC) face unique challenges obtaining high-quality, coordinated health care. With a growing number of veterans receiving VA-purchased community care (CC) provided outside the Veterans Health Administration (VA), evidence is needed on how veterans in this high-prevalence, marginalized subgroup experience CC.

**Objective:**

To compare experiences with CC over time for US veterans with and without MHC.

**Design, Setting, and Participants:**

This retrospective, cross-sectional survey study analyzed responses to the Survey of Healthcare Experiences of Patients–Community Care Survey (SHEP-CCS) from 2016 to 2021. Ratings of CC were examined across 9 domains and compared for veterans with and without MHC, adjusting for differences in baseline characteristics using regression models. Data were analyzed from March 2023 to September 2024.

**Exposure:**

Diagnosis of MHC, defined as bipolar disorder, major depression, posttraumatic stress disorder, schizophrenia, or psychosis.

**Main Outcomes and Measures:**

Veterans’ ratings of CC across 9 domains, overall satisfaction, overall clinician rating, clinician communication, eligibility determination, first appointment access, recent appointment access, nonappointment access, care coordination, and billing, were assessed on a scale of 1 to 100. Unadjusted annual ratings of care experiences were analyzed by survey domain. A series of 4 respondent-level linear regression models were examined for each domain and survey responses were pooled to test for differences in experiences between veterans with vs without MHC.

**Results:**

This study included 231 869 veterans, including 62 911 veterans with MHC (27.1%) and 168 958 without MHC (72.9%). Veterans with MHC had a mean (SD) age of 55.8 (14.7) years, 8327 were female (18.5%), and 24 792 had 3 or more comorbidities (29.9%). Veterans without MHC had a mean (SD) age of 62.5 (15.2) years, 11 277 were female (11.0%), and 49 689 had 3 or more comorbidities (24.0%). In fully adjusted models, veterans with vs without MHC had lower adjusted overall satisfaction with CC by −1.8 (95% CI, −2.3 to −1.3) points (*P* < .001). Ratings in all domains were lower for veterans with vs without MHC (−0.09 to −0.05 SDs of domain scores) (*P* < .001 for all comparisons). Although ratings improved from 2016 to 2021, significant differences persisted over time for veterans with vs without MHC for all domains.

**Conclusions and Relevance:**

In this survey study of veterans receiving CC from 2016 to 2021, those diagnosed with MHC reported lower ratings of CC across all measured domains, and these differences persisted over time. These findings highlight where focused care coordination and quality improvement efforts could improve CC experiences for this vulnerable subpopulation of veterans.

## Key Points

**Question:**

How do veteran experiences with Veterans Health Administration–purchased community care (CC) differ for veterans with vs without mental health conditions (MHC)?

**Findings:**

In this survey study of 231 869 US veterans surveyed from 2016 to 2021, those with vs without MHC reported significantly lower ratings of experiences with CC in 9 domains, including a 1.8-point lower overall satisfaction rating (0 to 100-point scale). Although ratings of CC improved over time, differences in experiences persisted between veterans with and without MHC.

**Meaning:**

These findings suggest that among veterans using CC, those with MHC may be less satisfied with CC, underscoring a need for targeted efforts to improve CC for this subpopulation of veterans.

## Teaser

This survey study compares experiences with community care purchased by the Veterans Health Administration (VA) over time for US veterans with and without mental health conditions.

## Introduction

More than 1 in 4 veterans have a diagnosis of a behavioral health condition, including a mental illness or substance use disorder.^1^ Veterans diagnosed with mental health conditions (MHC)—defined in this study as major depression, posttraumatic stress disorder, psychosis, schizophrenia, and bipolar disorder—are likely to have multiple medical and behavioral comorbidities and are at greater risk of hospitalization and mortality compared with veterans without MHC.^1,2,3^ Managing the unique needs of veterans with MHC requires coordination between numerous clinicians, placing an increased burden on individuals who are particularly susceptible to care fragmentation.^4,5^ However, veterans with MHC report greater difficulty receiving adequate health care coordination than veterans without MHC.^6,7^

The urgency of identifying and addressing barriers to high-quality care among veterans with MHC has increased with the introduction of the Veterans Choice Program in 2014 and the Maintaining Internal Systems and Strengthening Integrated Outside Networks Act (MISSION Act) in 2019.^8^ These policies expanded veterans’ eligibility for and access to VA-funded community care (CC) provided by clinicians and hospitals outside the VA health care system.^9^ Increases in the number of veterans receiving care both within and outside the VA have amplified concerns about the quality and coordination of care for vulnerable veterans, including those with MHC.^7,10,11,12^

Therefore, it is crucial to examine whether veterans with MHC experience greater challenges navigating CC and report poorer experiences with care provided by non-VA clinicians compared with veterans without MHC. Research has found that veterans seeking behavioral health care in the community frequently face challenges, such as long wait times to schedule first appointments and a lack of familiarity with military culture among clinicians based outside of the VA.^13^ However, national evidence comparing the experiences of veterans with and without MHC receiving CC remains limited.

This study examined veterans’ experiences with CC over time for those with and without a diagnosed MHC. We analyzed national data from the VA Survey of Healthcare Experiences of Patients—Community Care Survey (SHEP-CCS) from 2016 through 2021 to examine veterans’ self-reported experiences with community care in several domains, including interactions with clinicians, navigating eligibility, and experiences with care coordination.^14^

## Methods

The VA Pittsburgh Healthcare System Institutional Review Board determined this study to be exempt. This was secondary data analysis of deidentified data and did not require informed consent. Results reported adhere to the American Association for Public Opinion Research (AAPOR) reporting guideline. This study also followed the Strengthening the Reporting of Observational Studies in Epidemiology (STROBE) reporting guideline for cross-sectional studies.

### Data Sources

We analyzed the national VA Survey of Healthcare Experiences of Patients—Community Care Survey (SHEP-CCS) from 2016 to 2021. SHEP-CCS is a mixed-mode (internet and mail) survey of veterans who received CC within the prior 90 days.^15^ Respondents rated their experiences with CC in 9 domains, including clinician communication, care coordination, navigating eligibility determination, and billing.

We linked SHEP-CCS to 3 data sources: (1) VA Corporate Data Warehouse (CDW) files, which included VA Program Integrity Tool (PIT) files and VA Fee Basis files; (2) Medicare and Medicaid enrollment files from the Centers for Medicare & Medicaid Services (CMS); and (3) United States Veterans Eligibility Trends and Statistics (USVETS) data. VA CDW captured information about veterans’ priority group status (which reflects veterans’ income and level of service-connected disability) and diagnoses identified by VA clinicians. VA PIT files and Fee Basis files captured veterans’ diagnoses identified during community care visits. We used CMS data to determine whether veterans were enrolled in Medicare, Medicaid, or both programs. USVETS data were used to assess educational attainment.

We linked these data to county-level information from the Area Health Resources File^16^ to measure the supply of physicians (overall and psychiatrists), residence in a health professional shortage area, and residence in a rural vs urban area (based on urban influence codes). eTable 1 in Supplement 1 provides additional information about our data sources.

### Population and Sample

Our analysis included veterans surveyed by SHEP-CCS from 2016 to 2021 (eTable 2A in Supplement 1). From an initial sample of 233 634 respondents, we excluded individuals who could not be linked to VA CDW data (188 veterans [0.1%]), resided outside the 50 US states or Washington DC (1093 veterans [0.5%]) (eTable 2B in Supplement 1), and did not have geographic identifiers needed for linkage to county-level data (484 veterans [0.2%]). The final sample included 231 869 observations.

### Mental Health Condition

We identified veterans with MHC as those diagnosed with bipolar disorder, major depression, posttraumatic stress disorder, schizophrenia, or psychosis. The presence of these conditions was assessed from documented diagnoses in VA CDW, PIT, and Fee Basis data in the 2 years preceding the SHEP-CCS survey. We used an approach similar to the Elixhauser comorbidity algorithm (a commonly used method of identifying chronic conditions in administrative data)^17^ and required the presence of at least 1 inpatient diagnosis, or 2 outpatient diagnoses at least 30 days apart, to establish the presence of a condition (eTable 3 in Supplement 1). In our sample of veterans with MHC, 40 433 (64.3%) had major depression, 37 747 (60.0%) had posttraumatic stress disorder (PTSD), 4803 (7.6%) had bipolar disorder, 1807 (2.9%) had schizophrenia, and 1007 (1.6%) had psychosis (eTable 4 in Supplement 1).

### Outcomes

We assessed veterans’ self-reported experiences with CC based on responses to 33 SHEP-CCS items, which we aggregated into 9 domain scores using item-domain groupings developed by the VA (eTable 5 in Supplement 1). The domain scores captured veterans’ overall satisfaction with community care, overall clinician ratings, experiences navigating the eligibility determination process, first appointment access, timely access for a recent appointment, clinician communication, care coordination, nonappointment access (eg, after-hours access to clinicians and waiting time in the office), and billing. For each respondent, we calculated a domain score as the equally weighted average of the respondent’s ratings for items under the domain. Items were linearly transformed onto a 100-point scale before aggregation. Higher scores indicate greater satisfaction with care.

### Covariates

We adjusted for covariates capturing veterans’ demographic characteristics, health status, type of community care received, geography, insurance, and socioeconomic status. Demographic variables included age, gender, race, and ethnicity, all self-reported by respondents in the SHEP survey. Race and ethnicity data were assessed to examine social factors associated with racial and ethnic identity, which prior research has shown is linked to veterans’ experiences with VA CC.^14^ We controlled for the type of community care received by including indicators for medicine subspecialty care, surgical care, eye care, acupuncture, psychiatric care, primary care, and a residual other group.

We assessed health status using a continuous index based on 26 comorbidity indicators from the Elixhauser comorbidity index, excluding behavioral health conditions, as this study was explicitly focused on comparing veterans with and without MHC. We included a separate indicator denoting the diagnosis of a substance use disorder (defined as drug or alcohol use disorders), which we controlled for in our main analyses and used to stratify our sample in supplementary analyses.

Geographic variables included urbanicity, residence in a health professionals shortage area, the supply of physicians and psychiatrists per 1000 county residents, and indicators for VA Veterans Integrated Service Networks (VA administrative regions). Finally, to capture factors related to socioeconomic status, insurance, and disability, we included indicators for VA priority group status (categorized into three groups that reflect veterans’ income and level of service-connected disability),^18^ educational attainment, and insurance coverage through Medicaid, Medicare, Medicare Part D, and Medicare Advantage.

### Statistical Analyses

We conducted a retrospective, cross-sectional analysis of veterans’ responses to the SHEP from 2016 to 2021. We first analyzed unadjusted annual ratings of care experience by survey domain for veterans with vs without MHC. Next, for each domain, we estimated a series of 4 respondent-level linear regression models, pooling survey responses from 2016 to 2021, to test for differences in experiences between veterans with vs without MHC. We sequentially adjusted for covariates at each stage. Model 1 adjusted for demographic variables, type of community care received, and year fixed effects. Model 2 additionally adjusted for health status; model 3 added control variables for geography and county-level supply of clinicians; and model 4 additionally adjusted for measures of insurance, socioeconomic status, and disability. Estimating models in stages allowed us to examine the extent to which differences in care experiences may be mediated by factors, such as medical comorbidities, socioeconomic status, and health insurance, which differ among veterans with vs without MHC.^19,20^

In secondary analyses, we estimated logistic regression models to examine differences in the likelihood that veterans with MHC were more likely to rate their care highly or poorly than veterans without MHC. High ratings of care experience were defined by domain rating scores equivalent to or higher than the 90th percentile of the score distribution among all SHEP-CCS respondents in our sample across all study years. Low ratings were defined as scores less than or equal to the 10th percentile of the score distribution among all SHEP-CCS respondents in our sample across study years. For each domain score, we estimated a set of 4 models successively adjusting for covariates, with each model estimating the probability of a high or low rating of care as a function of MHC and covariates. To facilitate interpretation, we reported estimates from these models as marginal effects, representing adjusted percentage point differences in the probability of high and low care ratings.

Because the cooccurrence of MHC and substance use disorders may present distinct challenges for veterans receiving CC, we also examined whether experiences with CC differed according to the presence of MHC with or without SUD (defined as drug or alcohol use disorder). Specifically, we categorized veterans into 4 groups to distinguish those diagnosed with MHC and SUD, MHC alone, SUD alone, or neither condition, and analyzed annual CC ratings in these groups.

All analyses were conducted in Stata version 18 (StataCorp). Estimates were weighted using SHEP-CCS survey weights and robust standard errors were estimated. Statistical significance was assessed at *P* < .05. *P* values were not adjusted for multiple testing, as the outcomes were conceptually related, making corrections for independent hypothesis testing inappropriate. Data were analyzed from March 2023 to September 2024.

## Results

### Descriptive Statistics

Our sample included 231 869 person-year observations among veterans who used CC and responded to SHEP community care surveys between 2016 and 2021. This included 62 911 observations among veterans with MHC (weighted N, 5 145 551 person-years) and 168 958 observations among veterans without MHC (weighted N, 11 275 614 person-years) (Table). Veterans with MHC had a mean (SD) age of 55.8 (14.7) years and 8327 were female (18.5%), while those without MHC had a mean (SD) age of 62.5 (15.2) years and 11 277 were female (11.0%). Veterans with MHC vs those without MHC were more likely to have been diagnosed with a substance use disorder (9361 [15.1%] vs 5448 [3.1%]), have 3 or more Elixhauser comorbidities (24 792 [29.9%] vs 49 689 [24.0%]), have a VA priority group status of 1 to 4, indicating higher levels of service-connected disability (51 884 [86.2%] vs 95 250 [63.2%]), and have Medicaid insurance (4696 [8.8%] vs 9997 [6.1%]).

**Table.** Characteristics of Veterans in the Survey of Healthcare Experiences of Patients–Community Care Survey (SHEP-CCS) Survey Administered to Community Care Recipients, 2016 to 2021

Characteristic^a^	Diagnosed with a mental health condition (n = 62 911)^b^	Not diagnosed with a mental health condition (n = 168 958)	*P *value^c^
Weighted sample^d^	5 145 551	11 275 614	
Demographics			
Age, mean (SD), y	55.8 (14.7)	62.5 (15.2)	<.001
Female gender	8327 (18.5)	11 277 (11.0)	<.001
Ethnicity^e^			
Hispanic	5835 (11.0)	10 655 (7.6)	<.001
Non-Hispanic	52 634 (81.1)	148 091 (85.8)	
Unknown	4442 (7.9)	10 212 (6.6)	
Race^e^			
Black or African American	8594 (15.1)	15 712 (11.1)	<.001
White	45 238 (68.8)	135 075 (76.7)	
Other/missing^f^	9079 (16.2)	18 171 (12.2)	
Health status			
Substance use disorder^g^	9361 (15.1)	5448 (3.1)	<.001
Modified Elixhauser comorbidities^h^			
0	10 967 (27.5)	43 223 (33.7)	<.001
1-2	27 152 (42.5)	76 046 (42.3)	
>3	24 792 (29.9)	49 689 (24.0)	
Insurance and socioeconomic status			
VA priority group status^i^			
Group 1-4	51 884 (86.2)	95 250 (63.2)	<.001
Group 5	7831 (9.6)	39 288 (19.2)	
Group 6-8	3145 (4.2)	34 298 (17.5)	
Missing	51 (0.1)	122 (0.1)	
Enrolled in Medicaid	4696 (8.8)	9997 (6.1)	<.001
Enrolled in Medicare^j^	47 927 (56.7)	134 916 (60.9)	<.001
Enrolled in a Medicare Part D plan	11 849 (13.4)	41 194 (18.6)	<.001
Enrolled in a Medicare advantage plan	9814 (11.0)	33 947 (15.6)	<.001
Education			
Less than high school	2744 (2.7)	9945 (4.0)	<.001
High school	15 613 (20.0)	48 957 (24.9)	
College or more	42 193 (72.0)	105 183 (67.2)	
Missing	2361 (5.3)	4873 (3.9)	
Urbanicity^k^			
Large metropolitan area	15 895 (28.0)	37 054 (24.7)	<.001
Small metropolitan area	19 255 (31.8)	48 830 (29.8)	
Micropolitan area	13 768 (20.6)	38 952 (22.0)	
Rural area	13 993 (19.5)	44 122 (23.6)	
No. of physicians per 1000 county residents, mean (IQR)	1.9 (1.1-2.9)	1.8 (1.0-2.8)	<.001
No. of psychiatrists per 1000 county residents, mean (IQR)	0.1 (0.0-0.1)	0.1 (0.0-0.1)	<.001
Health professionals shortage area			
No	57 656 (91.9)	154 005 (91.6)	.13
Yes	5255 (8.1)	14 953 (8.4)	
Type of community care received^l^			
Surgical care	13 903 (20.6)	37 738 (22.2)	<.001
Medicine subspecialty	17 588 (20.0)	53 157 (24.1)	
Eye care	6023 (9.0)	24 177 (15.1)	
Acupuncture	14 957 (32.0)	28 642 (26.8)	
Psychiatric	4870 (12.5)	1566 (2.1)	
Primary care	1817 (2.3)	7949 (3.9)	
Other	3753 (3.7)	15 729 (5.7)	

Abbreviation: VA, Department of Veterans Affairs.

^a^ Table presents survey-weighted means (continuous variables) and proportions (categorical variables) of respondents to the VA SHEP-CCS, pooled from 2016 to 2021. Survey weighting accounts for sampling probability and nonresponse.

^b^ Mental health condition (MHC) was defined by a diagnosis of bipolar disorder, major depression, posttraumatic stress disorder, schizophrenia, or psychosis. Diagnoses were captured from the VA Corporate Data Warehouse, VA Program Integrity Tool file, and VA Fee Basis data for veterans during the 2 federal fiscal years preceding response to SHEP-CCS.

^c^ *t* test or Wilcoxon rank-sum test; χ^2^ test with Rao and Scott second-order correction.

^d^ Weighted sample sizes were calculated using SHEP-CCS survey weights.

^e^ Race and ethnicity self-reported by Veterans in the SHEP-CCS survey. Individuals categorized by Hispanic ethnicity included all racial groups, and individuals categorized by race included all ethnicities. Race and ethnicity information were obtained from respondents in SHEP-CCS.

^f^ The other/missing group includes individuals who identified their race as: American Indian or Alaska Native, Native Hawaiian or other Pacific Islander, Asian, other, or not reported.

^g^ Diagnoses of substance use disorder associated with drug or alcohol use were captured from the VA Corporate Data Warehouse, VA Program Integrity Tool file, and VA Fee Basis data for Veterans during the two federal fiscal years preceding response to SHEP-CCS.

^h^ Modified Elixhauser comorbidities includes 26 indicators that comprise the Elixhauser comorbidity index excluding *ICD-10-CM* codes for psychosis, depression and drug and alcohol abuse. Comorbidities were assessed from veterans’ diagnoses during the 2 federal fiscal years preceding response to SHEP-CCS and were extracted from the VA Corporate Data Warehouse, VA Program Integrity Tool file, and VA Fee Basis data.

^i^ Veterans are categorized by the VA into 1 of 8 priority groups based on military service, disability, income, and other benefit factors. Priority groups 1 to 4 are composed of veterans with the most significant levels of service-connected disability. Priority group 5 includes veterans with low incomes without service-connected disabilities. Priority group 6 encompasses veterans seeking care for radiation, toxic substance, or other environmental exposures. Priority groups 7 to 8 comprise veterans with nonservice-connected disabilities who are required to make copayments for VA care.

^j^ Includes individuals enrolled in traditional (ie, fee-for-service) Medicare and Medicare Advantage.

^k^ Urbanicity assessed from urban influence codes linked to veterans by county of residence.

^l^ VA SHEP-CCS respondents were stratified by the type of community care received during the prior 90 days. Our data are aggregated into 7 categories that broadly differentiate between types of community care received.

As shown in Table, veterans with MHC represented in our SHEP-CC sample used CC for various services: 13 903 received surgical care (20.6%), 17 588 used other medical subspecialty care (20.0%), and 4870 used psychiatric care in the community (12.5%). Among veterans without a diagnosed MHC, the most common types of community care used included acupuncture (28 642 [26.8%]), medical subspecialty care (53 157 [24.1%]), and surgical care (37 738 [22.2%]). The mean (SD) ratings with CC experiences for the full sample pooled across study years are available in eTable 6 in Supplement 1.

### Unadjusted Trends in Ratings of CC

**Figure 1.**
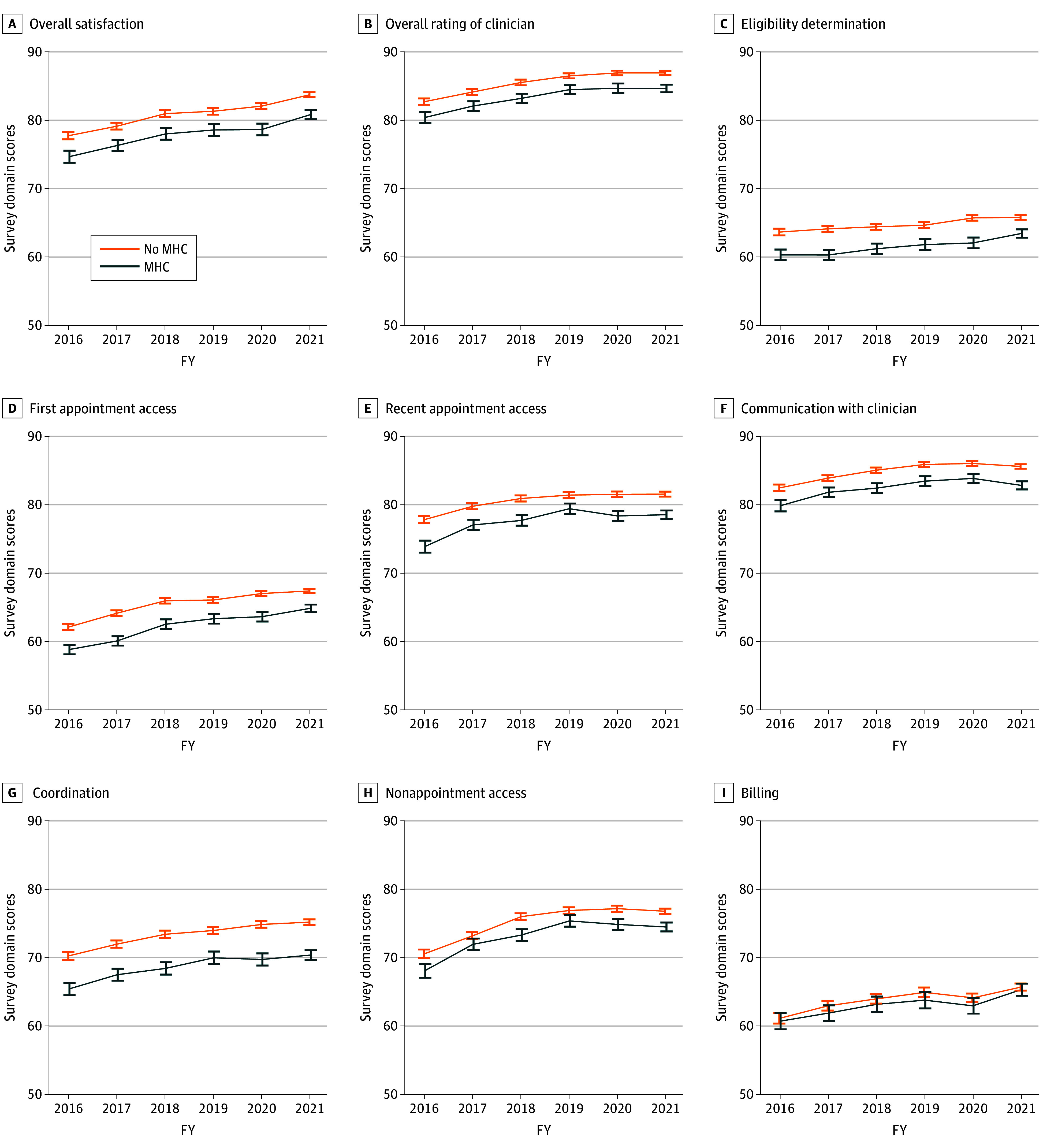
Unadjusted Annual Ratings of VA Community Care Experiences for Veterans With and Without Mental Health Condition (MHC) by Survey Domain, 2016-2021 Estimates from the VA Survey of Healthcare Experiences of Patients–Community Care Survey (SHEP-CCS). Estimates weighted using SHEP survey weights. Each panel represents ratings of community care in a particular domain. Domain scores calculated as the equally weighted average of the respondent’s ratings for items under the specified domain (transformed onto a consistent 0 to 100-point scale). Higher scores indicate greater satisfaction with care. FY indicates federal fiscal year.

In unadjusted analyses, veterans with MHC reported worse CC experiences than those without MHC in 8 of 9 survey domains (Figure 1). In each study year, veterans with MHC vs those without MHC reported lower overall satisfaction with CC, ratings of their community clinician, navigating eligibility determinations, scheduling a first appointment, recent appointment access, clinician communication, care coordination, and access to clinicians outside of scheduled appointments. Although ratings of CC experience improved over time, differences between veterans with and without MHC persisted. Furthermore, both veterans with and without MHC reported relatively worse experiences navigating eligibility determination, scheduling a first appointment, and billing compared with other domains.

In supplementary analyses, we found that differences in care ratings were most pronounced between veterans with and without MHC rather than between subgroups defined based on a diagnosis of MHC or SUD (ie, veterans with both MHC and SUD, MHC alone, SUD alone, or neither condition) (eFigure 1 in Supplement 1). Therefore, our primary analyses focused on the differences between veterans with vs without MHC.

### Adjusted Differences in Experiences With CC

**Figure 2.**
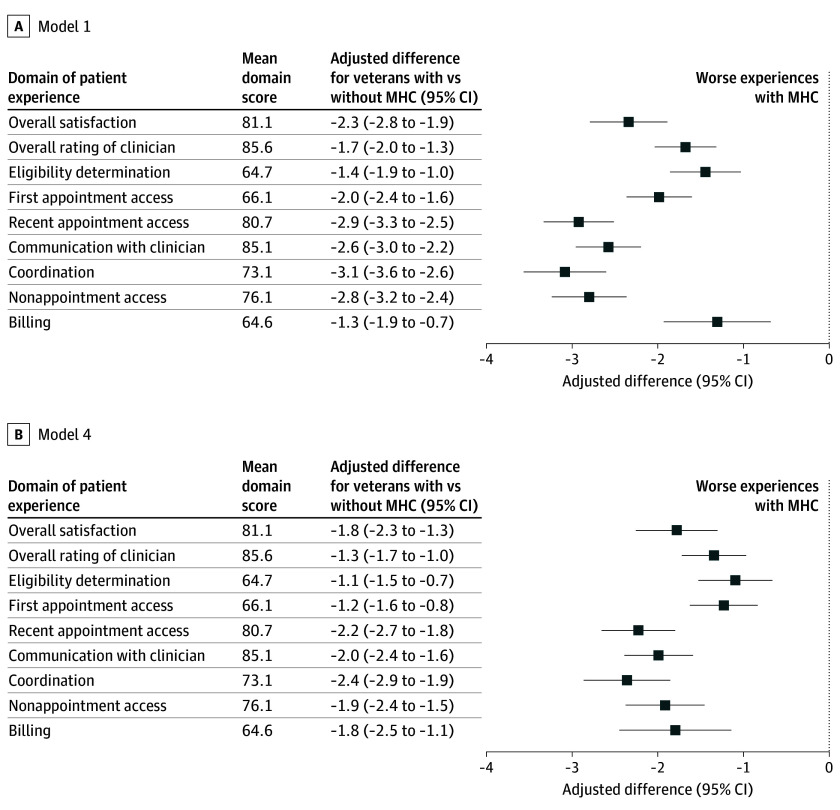
Adjusted Differences in Experiences With VA Community Care for Veterans With vs Without Mental Health Condition (MHC) by Survey Domain, 2016-2021 Plots show the differences in Survey of Healthcare Experiences of Patients–Community Care Survey (SHEP-CCS) survey domain score ratings, of community care experiences of veterans with and without MHC. Estimates use pooled data from 2016 to 2021. Adjusted difference estimated from a respondent-level linear regression model that estimated each domain score as a function of MHC, adjusting for covariates and year fixed effects. Adjusted differences are linear differences on a 100-point scale. Estimates weighted using SHEP-CCS survey weights. Bars indicate 95% CIs. Model 1 is adjusted for demographics and type of community care received. Model 4 is adjusted for demographics; type of community care received; health status; geography; and factors related to socioeconomic status, insurance, and disability. Estimates from Models 2 and 3 are shown in eFigure 2 in Supplement 1.

In adjusted analyses pooling study years, veterans with MHC reported significantly lower ratings of CC experiences across all 9 domains, compared with veterans without MHC (Figure 2 and eTable 7 in the Supplement). For example, in Model 1, veterans with MHC reported lower overall satisfaction with CC (−2.3 [95% CI, −2.8 to −1.9] points) compared with veterans without MHC. Expressed as an effect size (ie, adjusted difference divided by the SD of the domain score), this difference corresponded to an effect size of −0.09 SD. Veterans with MHC vs without MHC also reported lower ratings in other domains, including recent appointment access (−2.9 [95% CI, −3.3 to −2.5] points), ratings of clinician communication (−2.6 [95% CI, −3.0 to −2.2] points), and care coordination (−3.1 [95% CI, −3.6 to −2.6] points), corresponding to effect sizes of −0.12, −0.12, and −0.11 SDs, respectively.

Consistent with estimates from model 1, adjusted ratings of CC in model 4—our fully adjusted model—were lower in all domains among veterans with vs without MHC. For example, veterans with vs without MHC reported lower overall satisfaction with CC (−1.8 [95% CI, −2.3 to −1.3] points), recent appointment access (−2.2 [95% CI, −2.7 to −1.8] points), clinician communication (−2.0 [95% CI, −2.4 to −1.6] points), and care coordination (−2.4 [95% CI, −2.9 to −1.8] points) (Figure 2 and eTable 7 in Supplement 1). Across all survey domains, veterans with vs without MHC reported worse experiences with CC, with effect sizes for the differences ranging from −0.09 to −0.05 SDs of survey domain scores. These differences were all statistically significant at *P* < .001. Findings were similar in models 2 and 3 (eFigure 2 in Supplement 1).

### Adjusted Differences in the Probability of Reporting High vs Low Ratings of CC

**Figure 3.**
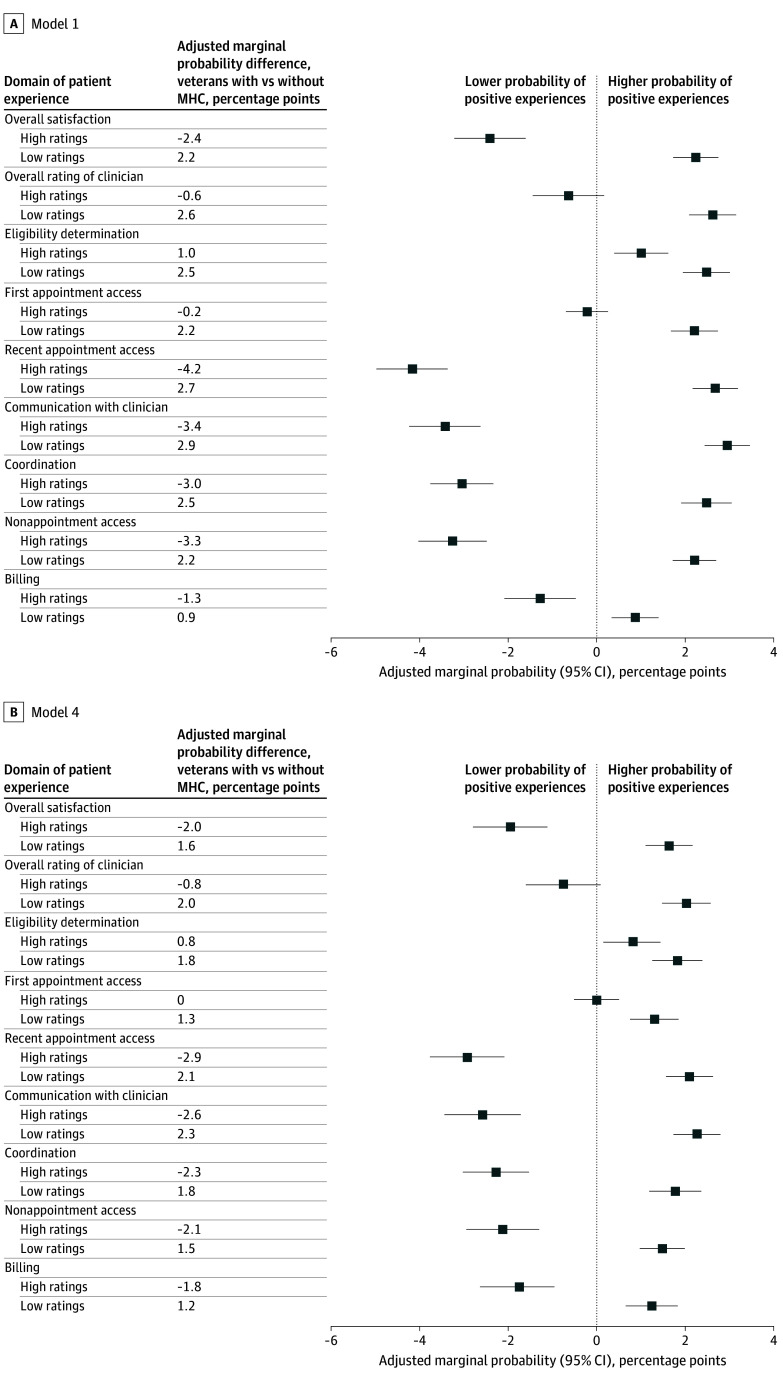
Adjusted Marginal Probability Differences for Positive and Negative Experiences With VA Community Care in Veterans With vs Without Mental Health Condition (MHC) by Survey Domain, 2016-2021 Plots show adjusted marginal differences in the probability that veterans with MHC report positive or negative community care experiences, relative to veterans without MHC. Estimates use pooled data from 2016 to 2021. Estimates from a respondent-level logistic regression model that estimated positive or negative ratings as a function of MHC, adjusting for covariates and year fixed effects. Marginal effects are reported, which reflect the difference in the probability (0-100 percentage point scale) of reporting positive or negative experiences between veterans with vs without MHC. Bars indicate 95% CIs. Positive experiences defined as ratings equal to or higher than the 90th percentile of the domain score distribution (among all Survey of Healthcare Experiences of Patients-Community Care Survey [SHEP-CCS] survey respondents in our sample and study years). Negative experiences defined as ratings equal to or lower than the 10th percentile of the distribution of the domain score (among all SHEP-CCS survey respondents in our sample and study years). Model 1 is adjusted for demographics and type of community care received. Model 4 is adjusted for demographics; type of community care received; health status; geography; and factors related to socioeconomic status, insurance, and disability. Estimates from models 2 and 3 are shown in eFigure 2 in Supplement 1.

Analyses from logistic regression models revealed veterans with MHC were more likely to report low CC ratings compared with veterans without MHC in all 9 survey domains and less likely to report high ratings of care in 7 of 9 domains (Figure 3). For example, in model 1, veterans with MHC vs without MHC were 2.2 (95% CI, 1.7 to 2.7) percentage points more likely to report low overall ratings of CC and 2.4 (95% CI, 1.6 to 3.2) percentage points less likely to report high ratings of CC. Estimated differences in the probability of reporting high and low ratings of CC were similar in Models 2 and 3 (eFigure 3 in Supplement 1) and Model 4 (Figure 3).

## Discussion

This national study of veterans receiving VA-purchased community care (CC) from 2016 to 2021 found that veterans diagnosed with MHC rated their experiences with CC lower than veterans without MHC. Although effect sizes were modest, with differences in ratings reported by veterans vs without MHC ranging from −0.05 to −0.09 SDs of domain scores in the fully adjusted models, these differences were statistically significant across all 9 survey domains and were largely consistent when examining the probability of reporting high and low ratings. Despite improvements in CC ratings over time, the gap in experiences between veterans with and without MHC persisted across all study years. These findings underscore the challenges vulnerable veterans experience when navigating and receiving community care and highlight an opportunity for targeted quality and care coordination strategies to improve CC for these veterans.

This study examined veterans’ experiences with community care, which encompassed a range of physical and behavioral health services. Notably, psychiatric care was only 12.5% of the community care services used by veterans with MHC, while services that included surgical care accounted for more than 20% of community care use among veterans with MHC. Prior research suggests that MHC increases the risk of care fragmentation and exacerbates the challenges of navigating both behavioral and physical health care.^6,21^ Our findings, in the context of this prior research, highlight the importance of providing holistic care coordination for veterans with MHC who use CC. The VA has existing programs to coordinate care for veterans, including the Referral Coordination Initiative, which uses Referral Coordination Teams at VA sites to discuss VA and CC care decisions with veterans.^22^ To identify veterans needing a higher level of care coordination, the VA uses metrics including the Care Assessment Need score, which estimates a veteran’s risk of hospitalization or death over the following year. Our findings suggest that the VA could explicitly consider MHC status in identifying veterans who may need additional care coordination. MHC status is already considered in VA-direct care, where programs, such as the Primary Care-Mental Health Integration (PC-MHI) model, provide enhanced care coordination for veterans with mental health conditions.^23^ Given the range of CC services veterans use, it may also be important to consider the need for care coordination even when veterans with MHC are not using CC for behavioral health care. Future research could help to identify where targeting additional care coordination resources in CC may yield the greatest benefit.

Prior literature has found that veterans with vs without behavioral health conditions report worse overall experiences with VA-direct care,^24,25,26^ and the current study is the first to identify this difference within CC. Our findings complement another study that compared veteran experiences in VA-direct vs CC care, which found lower scores in communication, coordination, and clinician ratings for CC vs VA-direct care across primary, mental health, and specialty care.^27^ Taken together, this evidence raises the question of how to manage CC where there is added complexity due to concomitant behavioral health or military cultural competence needs. The VA has a unique approach to integrating physical and behavioral health care and training clinicians in military cultural competency. However, non-VA community clinicians are not required to have this training and may not have many veteran patients, which may limit community clinicians’ preparedness to address the combined physical, behavioral, and military-specific needs of veterans with MHC. As the VA considers what type of care is reasonably retained in the VA vs provided through community partners, and what resources are needed to coordinate care across different systems, additional consideration should be given to vulnerable populations, including veterans with MHC.

### Limitations

This study had several limitations. First, our findings describe differences in CC experiences between veterans with and without MHC but cannot be interpreted as causal relationships. Second, because the SHEP-CCS was administered to veterans who used community care, we could not examine the experiences of veterans who sought but were unable to obtain CC. Consequently, we could not study barriers to CC access that may differ for veterans with MHC. Third, nonresponse to the SHEP-CCS could introduce bias, and the magnitude and direction of such bias is not known. Fourth, key analysis variables, such as diagnosis of an MHC, were assessed using administrative data and may be subject to measurement error. However, such measurement error would likely bias our estimates toward the null. Fifth, we did not include veterans in US territories, who were excluded due to differing CC eligibility criteria in these regions. Finally, we could not examine whether specific services contributed most to the lower ratings of care reported by veterans with MHC. SHEP-CCS measures broad constructs that are not specific to a type of care, and sample sizes were insufficient to stratify by type of CC service used by veterans. However, our analyses broadly illuminate the challenges veterans with MHC experience in CC.

## Conclusions

In this survey study of veterans using VA-funded CC during 2016 to 2021, veterans diagnosed with a MHC were found to have reported worse experiences with CC compared with veterans without a MHC. These findings underscore the need for ongoing efforts to enhance VA support for veterans with MHC who use VA-funded care in the community.

## Notes

Supplement 1. **eTable 1.** Data Sources and Information Used in This Study **eTable 2.** Sample Selection Criteria **eTable 3.** *ICD-9* and *ICD-10* Codes Included in Mental Health Condition (MHC) Diagnosis Category **eTable 4.** Behavioral Health Diagnoses Among Veterans in SHEP Sample With a Diagnosed Mental Health Condition (MHC), 2016 to 2021 **eTable 5.** Selected SHEP Questions and Response Scales Accessing Veterans’ Community Care Experiences Categorized by Care Experience Domain **eTable 6.** Descriptive Sample Statistics by Domain, Full Sample of Veterans, 2016 to 2021 **eFigure 1.** Unadjusted Annual Ratings of VA Community Care Experiences for Veterans With MHC, SUD, MHC and SUD, and Without MHC or SUD by Survey Domain, 2016 to 2021 **eTable 7.** Regression Model Outputs for Adjusted Differences in Experiences With VA Community Care for Veterans With vs Without MHC by Survey Domain, 2016 to 2021 **eFigure 2.** Adjusted Differences in Experiences With VA Community Care for Veterans With vs Without MHC by Survey Domain, 2016 to 2021 **eFigure 3.** Adjusted Marginal Probability Differences for Positive and Negative Experiences With VA Community Care in Veterans With vs Without MHC by Survey Domain, 2016 to 2021

Supplement 2.
Data Sharing Statement
