# Supplementary material for: Experiences With VA-Purchased Community Care for US Veterans With Mental Health Conditions
Source: JAMA Netw Open. 2025 May 21;8(5):e2511548. doi: 10.1001/jamanetworkopen.2025.11548 (PMC12096262; doi:10.1001/jamanetworkopen.2025.11548)
Supplement: Supplement 1. — eTable 1. Data Sources and Information Used in This Study eTable 2. Sample Selection Criteria eTable 3. ICD-9 and ICD-10 Codes Included in Mental Health Condition (MHC) Diagnosis Category eTable 4. Behavioral Health Diagnoses Among Veterans in SHEP Sample With a Diagnosed Mental Health Condition (MHC), 2016 to 2021 eTable 5. Selected SHEP Questions and Response Scales Accessing Veterans’ Community Care Experiences Categorized by Care Experience Domain eTable 6. Descriptive Sample Statistics by Domain, Full Sample of Veterans, 2016 to 2021 eFigure 1. Unadjusted Annual Ratings of VA Community Care Experiences for Veterans With MHC, SUD, MHC and SUD, and Without MHC or SUD by Survey Domain, 2016 to 2021 eTable 7. Regression Model Outputs for Adjusted Differences in Experiences With VA Community Care for Veterans With vs Without MHC by Survey Domain, 2016 to 2021 eFigure 2. Adjusted Differences in Experiences With VA Community Care for Veterans With vs Without MHC by Survey Domain, 2016 to 2021 eFigure 3. Adjusted Marginal Probability Differences for Positive and Negative Experiences With VA Community Care in Veterans With vs Without MHC by Survey Domain, 2016 to 2021 [file jamanetwopen-e2511548-s001.pdf]

Supplemental Online Content

Vanneman ME, Roberts ET, Li Y, et al.Experiences with VA-purchased community care for US veterans with mental health conditions. *JAMA Netw Open*. 2025;8(5):e2511548. doi:10.1001/jamanetworkopen.2025.11548

eTable 1. Data Sources and Information Used in This Study

eTable 2. Sample Selection Criteria

eTable 3. *ICD-9* and *ICD-10* Codes Included in Mental Health Condition (MHC) Diagnosis Category

eTable 4. Behavioral Health Diagnoses Among Veterans in SHEP Sample With a Diagnosed Mental Health Condition (MHC), 2016 to 2021

eTable 5. Selected SHEP Questions and Response Scales Accessing Veterans' Community Care Experiences Categorized by Care Experience Domain

eTable 6. Descriptive Sample Statistics by Domain, Full Sample of Veterans, 2016 to 2021

eFigure 1. Unadjusted Annual Ratings of VA Community Care Experiences for Veterans With MHC, SUD, MHC and SUD, and Without MHC or SUD by Survey Domain, 2016 to 2021

eTable 7. Regression Model Outputs for Adjusted Differences in Experiences With VA Community Care for Veterans With vs Without MHC by Survey Domain, 2016 to 2021

eFigure 2. Adjusted Differences in Experiences With VA Community Care for Veterans With vs Without MHC by Survey Domain, 2016 to 2021

eFigure 3. Adjusted Marginal Probability Differences for Positive and Negative Experiences With VA Community Care in Veterans With vs Without MHC by Survey Domain, 2016 to 2021

This supplemental material has been provided by the authors to give readers additional information about their work.

© 2025 Vanneman ME et al. *JAMA Network Open*.

eTable 1 lists key data sources used in this study and information derived from each data source. These variables were linked at the Veteran level using an encrypted identifier (scrambled Social Security Number).

eTable 1: Data sources and information used in this study

Data Source	Main information derived from data source
**1)** The VA Survey of Healthcare Experiences of Patients – Community Care Survey (SHEP-CCS)	▪ Veterans' community care experiences<br>▪ Demographic data for age, sex, race, ethnicity<br>▪ Type of community care received
**2)** VA Corporate Data Warehouse (CDW)	▪ VA Priority Group status<br>▪ Diagnoses documented in records of VA health care visits (a)
**3)** VA Program Integrity Tool (PIT) files within the CDW	▪ Diagnoses documented in claims for VA community care visits (a)<br>▪ Use of VA outpatient community care covered under the Choice program
**4)** VA Planning System Support Group (PSSG) files within the CDW	▪ County of residence in a given fiscal year used for linking to AHRF
**5)** Medicare enrollment	▪ Identifies co-enrollment in Medicare and Medicaid
**6)** US Veterans Eligibility Trends & Statistics (USVETS)	▪ Used to assess educational attainment of Veterans
**7)** Area Health Resources File (AHRF)	▪ Supply of physicians (overall and psychiatrists)<br>▪ Residence in a health professional shortage area<br>▪ Urbanicity based on urban influence codes

(a) We used diagnoses documented in VA CDW, VA PIT files, and VA fee basis claims to ascertain substance use disorder, mental health condition, and construct the modified Elixhauser comorbidity index.

© 2025 Vanneman ME et al. *JAMA Network Open*.

eTable 2: Sample selection criteria

eTable 2A[a]**:** Stepwise inclusion criteria of Veteran respondents from SHEP

Step	Criteria	Observations Meeting Criteria
1	Individuals who were sampled for and responded to SHEP (FY 2016-21)	233,634
2	Individuals linked to CDW	233,446
3	Individuals living in the 50 states or DC (i.e., excluding territories)[b]	232,353
4	Individuals linked to county-level data[c]	231,869

eTable 2B: Territories from which Veterans were excluded for sample analysis

State Abbreviation	State
VI	Virgin Islands
AS	American Samoa
PH	Philippines
PR	Puerto Rico
GU	Guam
MP	Northern Mariana Islands

[a] The table shows the stepwise sample inclusion criteria and sample sizes for Veterans included in the study analysis.
[b] Refer to eTable 2B for a list of excluded territories.
[c] County geographic identifier in Veteran-level VA adminisrtrative data.

© 2025 Vanneman ME et al. *JAMA Network Open*.

eTable 3: ICD-9 and ICD-10 codes included in mental health condition (MHC) diagnosis category

Diagnosis Code	Diagnosis Label
29381	Psychotic disorder with delusions in conditions classified elsewhere
29382	Psychotic disorder with hallucinations in conditions classified elsewhere
29500	Simple type schizophrenia, unspecified
29501	Simple type schizophrenia, subchronic
29502	Simple type schizophrenia, chronic
29503	Simple type schizophrenia, subchronic with acute exacerbation
29504	Simple type schizophrenia, chronic with acute exacerbation
29505	Simple type schizophrenia, in remission
29510	Disorganized type schizophrenia, unspecified
29511	Disorganized type schizophrenia, subchronic
29512	Disorganized type schizophrenia, chronic
29513	Disorganized type schizophrenia, subchronic with acute exacerbation
29514	Disorganized type schizophrenia, chronic with acute exacerbation
29515	Disorganized type schizophrenia, in remission
29520	Catatonic type schizophrenia, unspecified
29521	Catatonic type schizophrenia, subchronic
29522	Catatonic type schizophrenia, chronic
29523	Catatonic type schizophrenia, subchronic with acute exacerbation
29524	Catatonic type schizophrenia, chronic with acute exacerbation
29525	Catatonic type schizophrenia, in remission
29530	Paranoid type schizophrenia, unspecified
29531	Paranoid type schizophrenia, subchronic
29532	Paranoid type schizophrenia, chronic
29533	Paranoid type schizophrenia, subchronic with acute exacerbation
29534	Paranoid type schizophrenia, chronic with acute exacerbation
29535	Paranoid type schizophrenia, in remission
29540	Schizophreniform disorder, unspecified
29541	Schizophreniform disorder, subchronic
29542	Schizophreniform disorder, chronic
29543	Schizophreniform disorder, subchronic with acute exacerbation
29544	Schizophreniform disorder, chronic with acute exacerbation
29545	Schizophreniform disorder, in remission
29550	Latent schizophrenia, unspecified
29551	Latent schizophrenia, subchronic
29552	Latent schizophrenia, chronic
29553	Latent schizophrenia, subchronic with acute exacerbation
29554	Latent schizophrenia, chronic with acute exacerbation

© 2025 Vanneman ME et al. *JAMA Network Open*.

Diagnosis Code	Diagnosis Label
29555	Latent schizophrenia, in remission
29560	Schizophrenic disorders, residual type, unspecified
29561	Schizophrenic disorders, residual type, subchronic
29562	Schizophrenic disorders, residual type, chronic
29563	Schizophrenic disorders, residual type, subchronic with acute exacerbation
29564	Schizophrenic disorders, residual type, chronic with acute exacerbation
29565	Schizophrenic disorders, residual type, in remission
29570	Schizoaffective disorder, unspecified
29571	Schizoaffective disorder, subchronic
29572	Schizoaffective disorder, chronic
29573	Schizoaffective disorder, subchronic with acute exacerbation
29574	Schizoaffective disorder, chronic with acute exacerbation
29575	Schizoaffective disorder, in remission
29580	Other specified types of schizophrenia, unspecified
29581	Other specified types of schizophrenia, subchronic
29582	Other specified types of schizophrenia, chronic
29583	Other specified types of schizophrenia, subchronic with acute exacerbation
29584	Other specified types of schizophrenia, chronic with acute exacerbation
29585	Other specified types of schizophrenia, in remission
29590	Unspecified schizophrenia, unspecified
29591	Unspecified schizophrenia, subchronic
29592	Unspecified schizophrenia, chronic
29593	Unspecified schizophrenia, subchronic with acute exacerbation
29594	Unspecified schizophrenia, chronic with acute exacerbation
29595	Unspecified schizophrenia, in remission
29600	Bipolar I disorder, single manic episode, unspecified
29601	Bipolar I disorder, single manic episode, mild
29602	Bipolar I disorder, single manic episode, moderate
29603	Bipolar I disorder, single manic episode, severe, without mention of psychotic behavior
29604	Bipolar I disorder, single manic episode, severe, specified as with psychotic behavior
29605	Bipolar I disorder, single manic episode, in partial or unspecified remission
29606	Bipolar I disorder, single manic episode, in full remission
29610	Manic affective disorder, recurrent episode, unspecified
29611	Manic affective disorder, recurrent episode, mild
29612	Manic affective disorder, recurrent episode, moderate
29613	Manic affective disorder, recurrent episode, severe, without mention of psychotic behavior
29614	Manic affective disorder, recurrent episode, severe, specified as with psychotic behavior
29615	Manic affective disorder, recurrent episode, in partial or unspecified remission

© 2025 Vanneman ME et al. *JAMA Network Open*.

Diagnosis Code	Diagnosis Label
29616	Manic affective disorder, recurrent episode, in full remission
29620	Major depressive affective disorder, single episode, unspecified
29621	Major depressive affective disorder, single episode, mild
29622	Major depressive affective disorder, single episode, moderate
29623	Major depressive affective disorder, single episode, severe, without mention of psychotic behavior
29624	Major depressive affective disorder, single episode, severe, specified as with psychotic behavior
29625	Major depressive affective disorder, single episode, in partial or unspecified remission
29626	Major depressive affective disorder, single episode, in full remission
29630	Major depressive affective disorder, recurrent episode, unspecified
29631	Major depressive affective disorder, recurrent episode, mild
29632	Major depressive affective disorder, recurrent episode, moderate
29633	Major depressive affective disorder, recurrent episode, severe, without mention of psychotic behavior
29634	Major depressive affective disorder, recurrent episode, severe, specified as with psychotic behavior
29635	Major depressive affective disorder, recurrent episode, in partial or unspecified remission
29636	Major depressive affective disorder, recurrent episode, in full remission
29640	Bipolar I disorder, most recent episode (or current) manic, unspecified
29641	Bipolar I disorder, most recent episode (or current) manic, mild
29642	Bipolar I disorder, most recent episode (or current) manic, moderate
29643	Bipolar I disorder, most recent episode (or current) manic, severe, without mention of psychotic behavior
29644	Bipolar I disorder, most recent episode (or current) manic, severe, specified as with psychotic behavior
29645	Bipolar I disorder, most recent episode (or current) manic, in partial or unspecified remission
29646	Bipolar I disorder, most recent episode (or current) manic, in full remission
29650	Bipolar I disorder, most recent episode (or current) depressed, unspecified
29651	Bipolar I disorder, most recent episode (or current) depressed, mild
29652	Bipolar I disorder, most recent episode (or current) depressed, moderate
29653	Bipolar I disorder, most recent episode (or current) depressed, severe, without mention of psychotic behavior
29654	Bipolar I disorder, most recent episode (or current) depressed, severe, specified as with psychotic behavior
29655	Bipolar I disorder, most recent episode (or current) depressed, in partial or unspecified remission
29656	Bipolar I disorder, most recent episode (or current) depressed, in full remission
29660	Bipolar I disorder, most recent episode (or current) mixed, unspecified
29661	Bipolar I disorder, most recent episode (or current) mixed, mild
29662	Bipolar I disorder, most recent episode (or current) mixed, moderate
29663	Bipolar I disorder, most recent episode (or current) mixed, severe, without mention of psychotic behavior
29664	Bipolar I disorder, most recent episode (or current) mixed, severe, specified as with psychotic behavior
29665	Bipolar I disorder, most recent episode (or current) mixed, in partial or unspecified remission

© 2025 Vanneman ME et al. *JAMA Network Open*.

Diagnosis Code	Diagnosis Label
29666	Bipolar I disorder, most recent episode (or current) mixed, in full remission
2967	Bipolar I disorder, most recent episode (or current) unspecified
29680	Bipolar disorder, unspecified
29681	Atypical manic disorder
29682	Atypical depressive disorder
29689	Other bipolar disorders
2971	Delusional disorder
2972	Paraphrenia
2973	Shared psychotic disorder
2978	Other specified paranoid states
2979	Unspecified paranoid state
2980	Depressive type psychosis
2981	Excitative type psychosis
2982	Reactive confusion
2983	Acute paranoid reaction
2984	Psychogenic paranoid psychosis
2988	Other and unspecified reactive psychosis
2989	Unspecified psychosis
3091	Prolonged depressive reaction
30981	Posttraumatic stress disorder
311	Depressive disorder, not elsewhere classified
F060	Psychotic disorder with hallucinations due to known physiological condition
F061	Catatonic disorder due to known physiological condition
F062	Psychotic disorder with delusions due to known physiological condition
F0631	Mood disorder due to known physiological condition with depressive features
F0632	Mood disorder due to known physiological condition with major depressive-like episode
F0633	Mood disorder due to known physiological condition with manic features
F0634	Mood disorder due to known physiological condition with mixed features
F200	Paranoid schizophrenia
F201	Disorganized schizophrenia
F202	Catatonic schizophrenia
F203	Undifferentiated schizophrenia
F205	Residual schizophrenia
F2081	Schizophreniform disorder
F2089	Other schizophrenia
F209	Schizophrenia, unspecified
F21	Schizotypal disorder
F22	Delusional disorders

© 2025 Vanneman ME et al. *JAMA Network Open*.

Diagnosis Code	Diagnosis Label
F23	Brief psychotic disorder
F24	Shared psychotic disorder
F250	Schizoaffective disorder, bipolar type
F251	Schizoaffective disorder, depressive type
F258	Other schizoaffective disorders
F259	Schizoaffective disorder, unspecified
F28	Other psychotic disorder not due to a substance or known physiological condition
F29	Unspecified psychosis not due to a substance or known physiological condition
F3010	Manic episode without psychotic symptoms, unspecified
F3011	Manic episode without psychotic symptoms, mild
F3012	Manic episode without psychotic symptoms, moderate
F3013	Manic episode, severe, without psychotic symptoms
F302	Manic episode, severe with psychotic symptoms
F303	Manic episode in partial remission
F304	Manic episode in full remission
F308	Other manic episodes
F309	Manic episode, unspecified
F310	Bipolar disorder, current episode hypomanic
F3110	Bipolar disorder, current episode manic without psychotic features, unspecified
F3111	Bipolar disorder, current episode manic without psychotic features, mild
F3112	Bipolar disorder, current episode manic without psychotic features, moderate
F3113	Bipolar disorder, current episode manic without psychotic features, severe
F312	Bipolar disorder, current episode manic severe with psychotic features
F3130	Bipolar disorder, current episode depressed, mild or moderate severity, unspecified
F3131	Bipolar disorder, current episode depressed, mild
F3132	Bipolar disorder, current episode depressed, moderate
F314	Bipolar disorder, current episode depressed, severe, without psychotic features
F315	Bipolar disorder, current episode depressed, severe, with psychotic features
F3160	Bipolar disorder, current episode mixed, unspecified
F3161	Bipolar disorder, current episode mixed, mild
F3162	Bipolar disorder, current episode mixed, moderate
F3163	Bipolar disorder, current episode mixed, severe, without psychotic features
F3164	Bipolar disorder, current episode mixed, severe, with psychotic features
F3170	Bipolar disorder, currently in remission, most recent episode unspecified
F3171	Bipolar disorder, in partial remission, most recent episode hypomanic
F3172	Bipolar disorder, in full remission, most recent episode hypomanic
F3173	Bipolar disorder, in partial remission, most recent episode manic
F3174	Bipolar disorder, in full remission, most recent episode manic

© 2025 Vanneman ME et al. *JAMA Network Open*.

Diagnosis Code	Diagnosis Label
F3175	Bipolar disorder, in partial remission, most recent episode depressed
F3176	Bipolar disorder, in full remission, most recent episode depressed
F3177	Bipolar disorder, in partial remission, most recent episode mixed
F3178	Bipolar disorder, in full remission, most recent episode mixed
F3181	Bipolar II disorder
F3189	Other bipolar disorder
F319	Bipolar disorder, unspecified
F320	Major depressive disorder, single episode, mild
F321	Major depressive disorder, single episode, moderate
F322	Major depressive disorder, single episode, severe without psychotic features
F323	Major depressive disorder, single episode, severe with psychotic features
F324	Major depressive disorder, single episode, in partial remission
F325	Major depressive disorder, single episode, in full remission
F329	Major depressive disorder, single episode, unspecified
F330	Major depressive disorder, recurrent, mild
F331	Major depressive disorder, recurrent, moderate
F332	Major depressive disorder, recurrent severe without psychotic features
F333	Major depressive disorder, recurrent, severe with psychotic symptoms
F3340	Major depressive disorder, recurrent, in remission, unspecified
F3341	Major depressive disorder, recurrent, in partial remission
F3342	Major depressive disorder, recurrent, in full remission
F339	Major depressive disorder, recurrent, unspecified
F4310	Post-traumatic stress disorder, unspecified
F4311	Post-traumatic stress disorder, acute
F4312	Post-traumatic stress disorder, chronic

© 2025 Vanneman ME et al. *JAMA Network Open*.

eTable 4: Behavioral health diagnoses among Veterans in SHEP sample with a diagnosed mental health condition (MHC), 2016-2021

Condition	N (%)<br>Total N=62,911
Post-Traumatic Stress Disorder (PTSD)	37,747 (60.0%)
Bipolar Disorder	4,803 (7.6%)
Major Depression	40,433 (64.3%)
Schizophrenia	1,807 (2.9%)
Psychosis	1,007 (1.6%)

Percentages sum to more than 100% because Veterans may be diagnosed with more than one behavioral health condition.

© 2025 Vanneman ME et al. *JAMA Network Open*.

eTable 5: Selected SHEP questions and response scales accessing Veterans' community care experiences categorized by care experience domain

Domain	Response Scale	Questions
Overall Satisfaction	Very dissatisfied, Dissatisfied, Somewhat dissatisfied, Somewhat satisfied, Satisfied, Very satisfied	Q40. Overall, how satisfied are you with your VA Community Care?
Overall Rating of Provider	0, 1, 2, 3, 4, 5, 6, 7, 8, 9, 10	Q35. Using any number from 0 to 10, where 0 is the worst provider possible and 10 is the best provider possible, what number would you use to rate your VA Community Care provider?
Eligibility Determination	Strongly disagree, Disagree, Neither agree nor disagree, Agree, Strongly agree	Q3. The eligibility requirements for VA Community Care are clear.<br>Q4. The information available about eligibility for VA Community care is helpful.
First Appointment Access	Strongly disagree, Disagree, Neither agree nor disagree, Agree, Strongly agree	Q5. The process for scheduling my first appointment for this service was clearly explained to me.<br>Q6. It was clear who was responsible for the process of arranging my first appointment for this service.<br>Q7. I had enough say in selecting a VA Community Care provider for this service.<br>Q8. I had enough say in selecting the date and time of my first appointment for this service.<br>Q9. I was able to get my first appointment for this service as soon as I needed.<br>Q10. It was easy to get my first appointment for this service.<br>Q11. I understand the process for getting VA Community Care, including determining eligibility, finding a community provider, and scheduling an appointment.
Recent Appointment Access	Never, Sometimes, Usually, Always	Q13. In the last 3 months, how often did you get an appointment for this service as soon as you needed?<br>Q14. In the last 3 months, how often were you able to get an appointment for this service at a convenient date and time?<br>Q15. In the last 3 months, how often were you able to receive this service at a convenient location?
Communication with Provider	Never, Sometimes, Usually, Always	Q17. In the last 3 months, how often did your VA Community Care provider explain things in a way that was easy to understand?<br>Q18. In the last 3 months, how often did your VA Community Care provider listen carefully to you?<br>Q20. In the last 3 months, how often did your VA Community Care provider give you easy to understand information about these health questions or concerns?<br>Q25. In the last 3 months, how often did your VA Community Care provider show respect for what you had to say?<br>Q26. In the last 3 months, how often did your VA Community Care provider spend enough time with you?
Coordination	Never, Sometimes, Usually, Always	Q21. In the last 3 months, how often did your VA Community Care provider seem to know the important information about your medical history?

© 2025 Vanneman ME et al. *JAMA Network Open*.

		Q22. In the last 3 months, how often did your VA Community Care provider seem informed and up-to-date about any care you received from VA providers?<br>Q23. In the last 3 months, how often did your VA provider(s) seem informed and up-to-date about your VA Community Care?<br>Q24. In the last 3 months, how often was it clear what the next step in your care would be?<br>Q28. In the last 3 months, when your VA Community Care provider ordered a blood test, x-ray or other test for you, how often did someone from your VA Community Care provider's office follow up to give you those results?<br>Q29. In the last 3 months, when your VA Community Care provider ordered a blood test, x-ray or other test for you, how often were the results also sent to the VA?
Non-appointment access	Never, Sometimes, Usually, Always	Q16. Wait time includes time spent in a waiting room and exam room. In the last 3 months, how often did you see your VA Community Care provider within 15 minutes of your scheduled appointment time?<br>Q30. In the last 3 months, when you contacted your VA Community Care provider's office during regular office hours, how often did you get an answer to your medical question that same day?<br>Q31. In the last 3 months, when you contacted your VA Community Care provider's office after regular office hours, how often did you get an answer to your medical question as soon as you needed?<br>Q33. In the last 3 months, when you contacted your VA Community Care provider's office using email, a web site or online tool, how often did you get a helpful response as soon as you needed?<br>Q34. In the last 3 months, when you phoned your VA Community Care provider's office, how often did you get a helpful response as soon as you needed?
Billing	Never, Sometimes, Usually, Always	Q36. In the last 3 months, how often was it clear whether or not you would have to make any out-of-pocket payments for your VA Community Care?<br>Q37. In the last 3 months, how often was the information about billing for VA Community Care clear?<br>Q39. In the last 3 months, how often has the process for handling bills for VA Community Care gone smoothly?

© 2025 Vanneman ME et al. *JAMA Network Open*.

eTable 6: Descriptive sample statistics by domain, full sample of Veterans, 2016-2021

Domain	Mean Rating	Standard Deviation of Rating
Overall Satisfaction	81.0793	25.1809
Overall Rating of Clinician	85.6313	20.6890
Eligibility Determination	64.7437	23.9157
First Appointment Access	66.1359	21.8679
Recent Appointment Access	80.6672	24.1132
Communication with Clinician	85.0534	21.7838
Coordination	73.0805	27.0262
Non-Appointment Access	76.0612	26.2220
Billing	64.5501	37.1858

Ratings are on a 0-100 scale, with higher ratings indicating greater satisfaction with care.

© 2025 Vanneman ME et al. *JAMA Network Open*.

eFigure 1: Unadjusted annual ratings of VA community care experiences for Veterans with MHC, SUD, MHC and SUD, and without MHC or SUD by survey domain, 2016-2021[abcd]

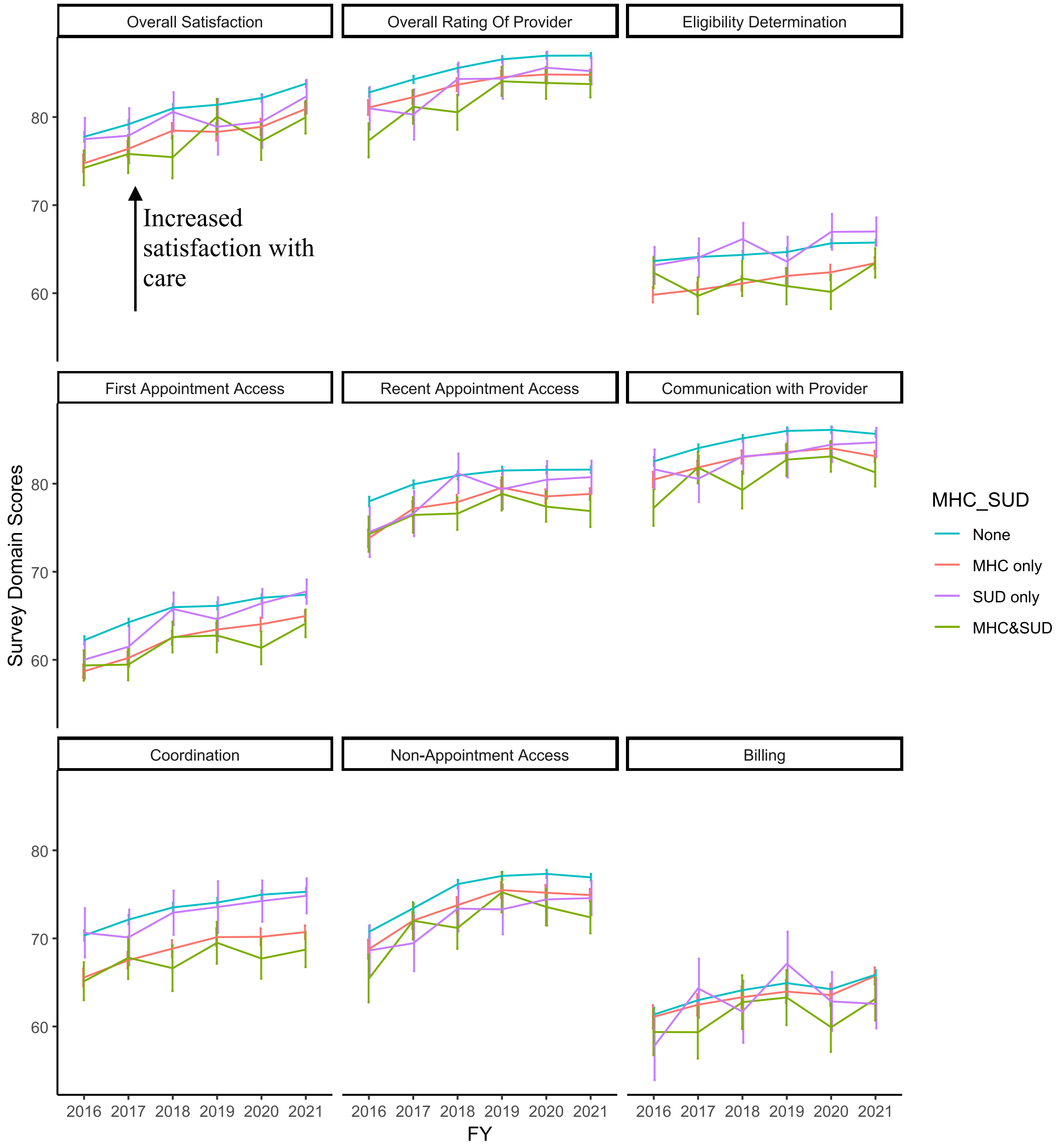

---

[a] Line graphs depict annual unadjusted mean score ratings of Veterans' experiences with community care in survey domain. Mean ratings are stratified by MHC versus SUD versus MHC and SUD versus no MHC or SUD. Estimates were weighted using survey weights. Bars indicate 95% confidence intervals using heteroskedacity-robust standard errors. Survey scores were linearly transformed onto a 100-point scale. FY is fiscal year.

[b] MHC was defined by diagnosis a of bipolar disorder, major depression, post-traumatic stress disorder, schizophrenia, or psychosis. Diagnoses from the two federal fiscal years preceding response to the SHEP survey were extracted from the VA corporate data warehouse or the VA Program Integrity Tool File.

[c] Diagnoses of SUD associated with drug or alcohol use were identified in the VA CDW and VA PIT files for Veterans during the two federal fiscal years preceding response to the SHEP survey.

[d] Red lines represent experience ratings of Veterans without MHC or SUD. Green lines represent experience ratings from Veterans with MHC. Purple lines represent experience ratings of Veterans with SUD. Blue lines represent experience ratings from Veterans with MHC and SUD.

eTable 7: Regression model outputs for adjusted differences in experiences with VA community care for Veterans with versus without MHC by survey domain, 2016-2021[a]

Model	Survey Domain	Adjusted Difference	p-value	Lower Confidence Interval	Upper Confidence Interval
1[b]	Overall Satisfaction	-2.34	<0.001	-2.79	-1.88
	Overall Rating of Clinician	-1.67	<0.001	-2.03	-1.32
	Eligibility Determination	-1.44	<0.001	-1.85	-1.03
	First Appointment Access	-1.98	<0.001	-2.36	-1.60
	Recent Appointment Access	-2.92	<0.001	-3.33	-2.51
	Communication with Clinician	-2.57	<0.001	-2.95	-2.19
	Coordination	-3.08	<0.001	-3.56	-2.60
	Non-Appointment Access	-2.80	<0.001	-3.23	-2.36
	Billing	-1.30	<0.001	-1.93	-0.68
2[c]	Overall Satisfaction	-2.17	<0.001	-2.64	-1.70
	Overall Rating of Clinician	-1.52	<0.001	-1.89	-1.15
	Eligibility Determination	-1.36	<0.001	-1.78	-0.94
	First Appointment Access	-1.75	<0.001	-2.14	-1.36
	Recent Appointment Access	-2.63	<0.001	-3.05	-2.21
	Communication with Clinician	-2.23	<0.001	-2.62	-1.84
	Coordination	-2.91	<0.001	-3.40	-2.41
	Non-Appointment Access	-2.19	<0.001	-2.64	-1.74
	Billing	-0.93	0.005	-1.57	-0.29
3[d]	Overall Satisfaction	-2.10	<0.001	-2.56	-1.63
	Overall Rating of Clinician	-1.46	<0.001	-1.83	-1.09
	Eligibility Determination	-1.36	<0.001	-1.79	-0.94
	First Appointment Access	-1.67	<0.001	-2.06	-1.28
	Recent Appointment Access	-2.55	<0.001	-2.97	-2.13
	Communication with Clinician	-2.17	<0.001	-2.56	-1.78
	Coordination	-2.82	<0.001	-3.31	-2.33
	Non-Appointment Access	-2.05	<0.001	-2.50	-1.60
	Billing	-0.88	0.007	-1.53	-0.24

[a] Adjusted differences represent the disparity in score ratings, aggregated from 2016 to 2021, of community care experiences for Veterans with and without MHC. Adjusted difference estimates from a respondent-level linear regression model that predicted each domain score as a function of MHC, adjusting for the varying covariates indicated in Table 1 (of the main manuscript) and year fixed effects. Refer to Methods and Table 1 for descriptions of the covariates. Adjusted differences are linear differences on a 100-point scale. Estimates were weighted using SHEP survey weights. 95% confidence intervals and p-values were calculated using heteroskedacity-robust standard errors. Lower Confidence Interval represents the lower bound of the 95% confidence interval and Upper Confidence Interval represents the upper bound of the 95% confidence interval.
[b] Model 1 is adjusted for demographics and type of community care received.
[c] Model 2 is adjusted for demographics; type of community care received; and health status.
[d] Model 3 is adjusted for demographics; type of community care received; health status; and geography.

© 2025 Vanneman ME et al. *JAMA Network Open*.

Model	Survey Domain	Adjusted Difference	p-value	Lower Confidence Interval	Upper Confidence Interval
4[a]	Overall Satisfaction	-1.77	<0.001	-2.25	-1.29
	Overall Rating of Clinician	-1.33	<0.001	-1.71	-0.96
	Eligibility Determination	-1.08	<0.001	-1.52	-0.65
	First Appointment Access	-1.22	<0.001	-1.62	-0.82
	Recent Appointment Access	-2.22	<0.001	-2.65	-1.79
	Communication with Clinician	-1.99	<0.001	-2.39	-1.58
	Coordination	-2.36	<0.001	-2.86	-1.85
	Non-Appointment Access	-1.91	<0.001	-2.37	-1.45
	Billing	-1.79	<0.001	-2.45	-1.13

[a] Model 4 is adjusted for demographics; type of community care received; health status; geography; and factors related to socioeconomic status, insurance, and disability.

© 2025 Vanneman ME et al. *JAMA Network Open*.

eFigure 2: Adjusted differences in experiences with VA community care for Veterans with versus without MHC by survey domain, 2016-2021[a]

Panel A: Model 2[b]

Domain of patient expereince	Average domain score	Adjusted difference	95% confidence interval
Overall Satisfaction	81.1	-2.2	
Overall Rating of Clinician	85.6	-1.5	
Eligibility Determination	64.7	-1.4	
First Appointment Access	66.1	-1.7	
Recent Appointment Access	80.7	-2.6	
Communication with Clinician	85.1	-2.2	
Coordination	73.1	-2.9	
Non-appointment Access	76.1	-2.2	
Billing	64.6	-0.9	

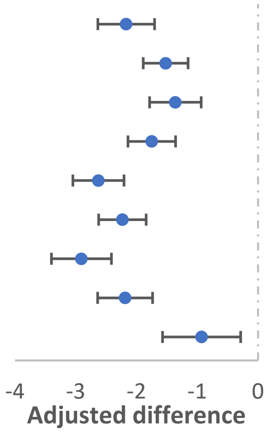

[a] Adjusted differences represent the disparity in score ratings, aggregated from 2016 to 2021, of community care experiences for Veterans with and without MHC. Adjusted difference estimates from a respondent-level linear regression model that predicted each domain score as a function of MHC, adjusting for the indicated covariates in Table 1 (of the main manuscript) and year fixed effects. Refer to Methods and Table 1 for descriptions of the covariates. Adjusted differences are linear differences on a 100-point scale. Estimates were weighted using SHEP survey weights. 95% confidence intervals were calculated using heteroskedacity-robust standard errors.
[b] Model 2 is adjusted for demographics; type of community care received; and health status.

Panel B: Model 3[a]

Domain of patient expereince	Average domain score	Adjusted difference	95% confidence interval
Overall Satisfaction	81.1	-2.1	
Overall Rating of Clinician	85.6	-1.5	
Eligibility Determination	64.7	-1.4	
First Appointment Access	66.1	-1.7	
Recent Appointment Access	80.7	-2.5	
Communication with Clinician	85.1	-2.2	
Coordination	73.1	-2.8	
Non-appointment Access	76.1	-2.0	
Billing	64.6	-0.9	

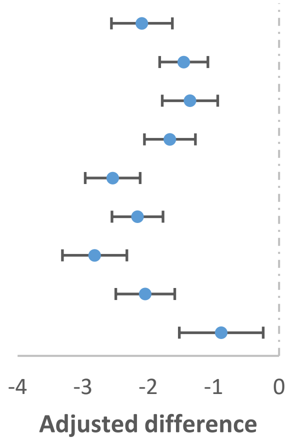

[a] Model 3 is adjusted for demographics; type of community care received; health status; and geography.

© 2025 Vanneman ME et al. *JAMA Network Open*.

eFigure 3. Adjusted marginal probability differences for positive and negative experiences with VA community care in Veterans with versus without MHC by survey domain, 2016-2021[abc]

Panel A: Model 2[d]

Domain of patient expereince	Adjusted maginal probability difference		95% confidence interval
	High ratings	*Low ratings*	
Overall Satisfaction	-2.1	2.2	
Overall Rating of Clinician	-0.7	2.4	
Eligibility Determination	0.9	2.3	
First Appointment Access	-0.1	2.0	
Recent Appointment Access	-3.6	2.5	
Communication with Clinician	-2.7	2.6	
Coordination	-2.6	2.4	
Non-appointment Access	-2.4	1.8	
Billing	-0.7	1.0	

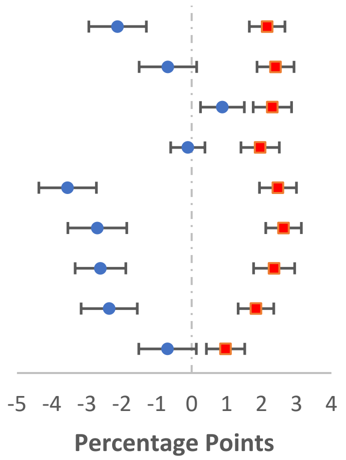

● High ratings ■ Low ratings

[a] The adjusted marginal probability differences represent the likelihood of Veterans reporting positive (blue dot) or negative (red dot) community care experiences, aggregated from 2016 to 2021, in Veterans with and without MHC. Disparity estimates are from a respondent-level logistic regression model that predicted each domain probability difference for positive or negative community care experiences as a function of MHC, adjusting for the varying covariates indicated in Table 1 (of the main manuscript) and year fixed effects. Refer to Methods and Table 1 for descriptions of the covariates. Adjusted differences are linear differences on a 100-point scale. Estimates were weighted using SHEP survey weights. 95% confidence intervals were calculated using heteroskedacity-robust standard errors.

[b] Positive experiences of care were defined by high ratings equivalent to or higher than the 90th percentile of the domain score distribution (among all SHEP survey respondents in our sample) and study years. Given the distribution of some scores is discrete, a rating equivalent to or higher than the 90th percentile may include more than 10% of Veterans.

[c] Negative experiences of care are defined by low ratings equivalent to or lower than the 10th percentile of the distribution of the domain score (among all SHEP survey respondents in our sample) and study years. Given the distribution of some scores is discrete, a rating equivalent to or lower than the 10th percentile may include more than 10% of Veterans.

[d] Model 2 is adjusted for demographics; type of community care received; and health status.

© 2025 Vanneman ME et al. *JAMA Network Open*.

Panel B: Model 3[a]

**Domain of patient expereince**	**Adjusted maginal probability difference**		**95% confidence interval**
	High ratings	*Low ratings*	
Overall Satisfaction	-2.1	2.2	
Overall Rating of Clinician	-0.8	2.4	
Eligibility Determination	0.9	2.3	
First Appointment Access	-0.1	1.9	
Recent Appointment Access	-3.6	2.4	
Communication with Clinician	-2.9	2.7	
Coordination	-2.7	2.4	
Non-appointment Access	-2.4	1.8	
Billing	-1.0	0.9	

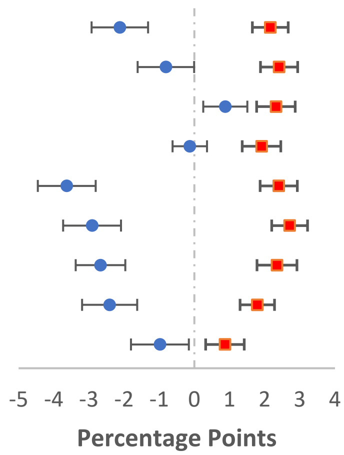

[a] Model 3 is adjusted for demographics; type of community care received; health status; and geography.

© 2025 Vanneman ME et al. *JAMA Network Open*.
