# Supplementary material for: Experiences With VA-Purchased Community Care for US Veterans With Mental Health Conditions
Source: JAMA Netw Open. 2025 May 21;8(5):e2511548. doi: 10.1001/jamanetworkopen.2025.11548 (PMC12096262; doi:10.1001/jamanetworkopen.2025.11548)
Supplement: Supplement 2. — Data Sharing Statement [file jamanetwopen-e2511548-s002.pdf]

Data Sharing Statement

Data
Data available: No

Additional Information
Explanation for why data not available: Use of these data is governed by a data use agreement with the Veterans Health Administration, which requires investigators to obtain approval to access the data. We will provide analytic code for our study online, so that an approved user of the data can replicate our analyses.
